# Practical methods to embed culture in dental education

**DOI:** 10.1038/s41415-026-0133-6

**Published:** 2026-10-09

**Authors:** K. Julia Hurry, Joelle Booth, Christopher Tredwin, Harriet Boyd

**Affiliations:** 784440818580552158573 Institute of Dentistry, Faculty of Medicine and Dentistry, Queen Mary University of London, London, United Kingdom; 538799616377091492834 Peninsula Dental School, University of Plymouth, Plymouth, United Kingdom; 439232151598304886351 School of Dentistry, University of Leeds, Leeds, United Kingdom

## Abstract

**Background** Culture is dynamic and multifaceted, influencing patients' beliefs, behaviours, and experiences of healthcare. In dentistry, a comprehensive understanding of culture, including social determinants, protected characteristics, and lived experience, is essential to avoid stereotyping and promote equitable care. The General Dental Council's *Safe practitioner framework* (*SPF*) integrates cultural competence across multiple domains of dental education.

**Aim** To explore how cultural competence (or humility) can be embedded into the UK dental curriculum in alignment with the *SPF*, and to offer practical examples to support educators.

**Methods** This narrative article utilises educational frameworks, current literature, and case examples from UK dental schools to illustrate methods for integrating cultural humility into teaching and learning.

**Results** Cultural humility can be embedded across domains, including clinical knowledge, clinical/technical skills, interpersonal skills, and professionalism. Educational strategies include case-based learning, reflective practice, community engagement, and the use of lived-experience resources. These approaches help students recognise how culture and social context influence disease presentation, communication, clinical decision-making, and patient engagement.

**Conclusion** Embedding cultural humility within dental education fosters the development of reflective, adaptable practitioners capable of delivering inclusive, patient-centred care. Alignment with the *SPF* provides an opportunity to enhance equity and responsiveness within the future dental workforce.

## Key points

- Cultural competence (or humility), underpinned by a broad understanding of culture, is essential for delivering equitable, patient-centred dental care in diverse populations.
- The General Dental Council's *Safe practitioner framework* embeds cultural competence across multiple domains, requiring integration throughout dental education rather than as a standalone topic.
- Practical educational approaches, such as reflective practice, community engagement, and lived-experience learning, support the development of adaptable, culturally responsive dental professionals.

## Background

### What is culture?

Culture is complex and multifaceted, extending beyond ethnicity or nationality. In his cornerstone paper, Hofstede (1980) described culture as ‘the collective programming of the mind which distinguishes the members of one group or category of people from another', emphasising its role in shaping values, beliefs and behaviours.^1^ More contemporary definitions recognise culture as being dynamic rather than static, acknowledging that individuals navigate multiple cultural identities simultaneously and that cultural expression evolves across contexts and generations.^2^

In healthcare contexts, culture encompasses not only visible elements such as language, diet or religious practices, but also deeper dimensions including communication styles, health beliefs, attitudes towards healthcare professionals, concepts of illness and wellness, and expectations of care.^3^ Betancourt *et al.* argue that a narrow view of culture focused solely on ethnic or racial categorisation risks stereotyping, whereas a broader understanding recognises the influence of socioeconomic status, education, sex, age, disability, sexual orientation and professional background in shaping an individual's worldview.^4^

For dental educators, this expanded definition has important implications. Students must be appropriately prepared to recognise their own assumptions, understand how power dynamics and institutional practices affect patient interactions, and develop the skills to negotiate healthcare encounters across different cultures.^5^ Rather than teaching students a prescriptive list of cultural ‘facts' about different groups, effective cultural education cultivates curiosity, humility and adaptable communication skills that can be applied across the diverse range of patient populations that dental professionals will encounter throughout their career.

### The *Safe practitioner framework*

In November 2023, the General Dental Council (GDC) introduced the *Safe practitioner framework* (*SPF*), which establishes the behaviours and outcomes expected of dental professional education across the UK.^6^ The *SPF* was developed through consultation with stakeholders including dental professionals, education and training providers and other professional bodies including charities and indemnity providers.^7^ Taking effect in August 2025, the GDC instructing that by the 2030/31 academic year, all undergraduate dental curricula in the UK should be mapped against the *SPF*.^6^

The *SPF* represents a significant evolution in dental education standards, explicitly embedding cultural competence (or humility) as a core component of safe practice across multiple domains.^8^ Within each dental profession's *SPF*, there are multiple learning outcomes on the importance of recognising the patient's cultural identity and how this can impact patient history, examination, diagnosis and management, as well as working as part of a culturally diverse dental team.^8^ Embedding cultural competency within dental education prepares dental professionals to develop the knowledge, skills and reflective capacity needed to practise with cultural humility and deliver inclusive, patient-centred care.^9^ Importantly, cultural humility is embedded across the continuum of dental education, from undergraduate training and early years dental training through to specialty training programmes^10^^,^^11^ This longitudinal approach enables developing practitioners to revisit and build upon their understanding at different stages, reinforcing key concepts and supporting the progressive development of culturally responsive practice.

The *SPF* is structured around four key domains:

- Clinical knowledge and skills
- Interpersonal skills
- Professionalism
- Self-management.

Cultural awareness and competence are woven throughout the framework, recognising that cultural competence is integral to all aspects of dental practice rather than an isolated skill set.^8^ This reinforces the expectation that cultural humility should be embedded within both the curriculum and the wider educational ethos.

The *SPF* signals a clear expectation that dental education must prepare graduates who not only possess clinical and technical proficiency, but who can deliver that care in ways that are responsive to diversity, attentive to structural inequalities and informed by patients' cultural contexts and lived experiences.

This article aims to provide practical tools to support dental educators in embedding culture within their curriculum, in line with the GDC's *SPF*.

## Culture-focused domains in the *Safe practitioner framework*

### Domain: Clinical knowledge and skills

#### *Sub-domain: Clinical knowledge and its application to patient management*

*C1.3: Explain the variance in disease presentation across diverse cultural and social groups, and those with protected characteristics, and how this impacts diagnosis, prevention, and treatment.*

Embedding cultural humility within clinical knowledge development (C1.3) requires dental professional trainees to recognise that oral disease does not present uniformly across populations, and that social, cultural and structural factors shape both disease patterns and patient experiences of care. This moves learning beyond biological determinism towards more nuanced, reflective understanding of patient management.

Trainees should be supported to understand how cultural practices, socioeconomic circumstances, lived experiences, and protected characteristics (as defined by the Equality Act 2010) influence oral health risks, access to care, symptom reporting and engagement with treatment.^12^ For example, oral hygiene and dietary practices linked to familial and cultural norms may affect caries risk,^13^ while experiences of discrimination or marginalisation may reduce trust in dental service and delay or prevent attendance.^14^ Cultural humility encourages learners to meet differences with curiosity, acknowledging the limits of their own knowledge and seeking out respectful dialogue that not only deepens understanding but also challenges preconceived biases by valuing others' lived experiences.

From a diagnostic perspective, disease presentation may be influenced by factors including skin tone, disability, gender identity, neurodiversity, or language barrier, which can affect clinical assessment, communication, and interpretation of symptoms. In prevention and treatment planning, cultural humility in practice involves adapting advice and interventions to be realistic, respectful and meaningful within the patient's social and cultural context.

Cultural humility within clinical knowledge can be embedded through curriculum-integrated teaching that links oral disease patterns to social determinants of health and protected characteristics, case-based learning that prompts reflection on how cultural and social context influences diagnosis and management, and reflective practice that encourages students to examine their assumptions and biases in clinical decision-making. The incorporation of lived experience through patient narratives, multimedia resources, and facilitated discussion can further support students in developing empathy, contextual understanding, and reflective practice. Examples of educational approaches aligned to this outcome are summarised in Table 1.

**Table 1:** Examples of educational initiatives that support the development of cultural humility within the GDC *Safe practitioner framework*

**Domain**	**Sub-domain**	**Examples**
Clinical knowledge and skills	Clinical knowledge and its application to patient management (C1.3)	Patient voice videos (University of Leeds): short videos co-produced with patients from groups with protected characteristics to explore lived experiences of healthcare Small-group discussion and reflection using patient narratives: facilitated discussion of patient experiences to promote empathy, perspective-taking and cultural humility Reflective journalling/debriefing: structured reflection following cultural humility teaching sessions Case-based learning: clinical cases exploring the influence of social and cultural context on diagnosis and management
	Clinical/technical skills (C2.1.1, C2.1.7, C2.2.1)	Community outreach learning (Bridge2Aid, University of Bristol): exposure to diverse patient groups and non-traditional care settings Humanising dentistry programme (University of Manchester): educational activities exploring patients' social circumstances alongside clinical needs Homeless community engagement initiatives: experiential learning with underserved populations to understand barriers to oral healthcare Community placements (University of Plymouth): student-led placements across diverse community settings
Interpersonal skills	Teamwork and wellbeing of others (I2.4)	EEDI curriculum resources (University of Glasgow): structured teaching on equity, equality, diversity and inclusion within the curriculum ‘Diversity Within Our Year' activities: student-led sharing of cultural identities and lived experiences Inclusive notice boards and communal displays: promotion of cultural awareness, wellbeing resources and inclusion initiatives Comic-strip microaggression resource (University of Dundee): reflective learning tool exploring experiences of microaggressions in healthcare education
Professionalism	Ethics and Integrity (P(B)3, P1.4)	World Café activities: Informal facilitated discussions exploring culture, identity and lived experience Co-produced film involving people with lived experience of the criminal justice system: educational resource exploring barriers to accessing dental care following imprisonment Inter-professional engagement module (Peninsula Dental School): community-based and interprofessional learning focused on local needs and health inequalities
EEDI, equality, equity, diversity and inclusion; IPE, inter-professional engagement.		

By meeting learning outcome C1.3, dental trainees are better equipped to deliver more accurate diagnoses by considering social and cultural influences on disease presentation, provide tailored preventative advice that aligns with patients' cultural practice and circumstances, develop person-centred treatment plans that improve engagement, adherence, and outcomes, and reduce inequalities in oral health by recognising and responding to structural and cultural barriers.

#### *Sub-domain: Clinical/technical skills*

*C2.1.1: Obtain, record, and interpret a comprehensive and contemporaneous patient history, taking into account social and/or cultural sensitivities*

*C2.1.7: Synthesise the full results of the patient's assessment and make clinical judgements taking into account patient compliance, values, cultural identity, and self-care*

*C2.2.1: Identify, explain, and manage the impact of medical and psychological conditions in a range of patients and determine how this can influence patient compliance, self-care, and outcomes, taking into account the patient's cultural identity and values.*

Embedding cultural humility within clinical and technical skills requires dental trainees to move beyond procedural competence and apply cultural humility consistently throughout patient assessment, decision-making, and care planning. This sub-domain emphasises not only technical competence, but the ability to apply clinical skills in ways that are responsive, ethical, and realistic for diverse patient populations.

Obtaining and recording a comprehensive patient history (C2.1.1) is inherently shaped by cultural and social context. Dental trainees should be supported in learning that patients may communicate symptoms differently depending on language, cultural norms, stigma, trauma, or prior experiences of healthcare.^15^^,^^16^ Similarly, self-reported pain in dental settings has been associated with gender-based cultural expectations around the expression of pain as an indication of weakness.^17^^,^^18^ Social factors such as housing insecurity, asylum status, disability, or experiences of discrimination may influence what patients feel safe to disclose and how they prioritise oral health.^14^^,^^19^ Cultural humility in history taking involves creating a non-judgemental space, using open-ended questioning, and recognising power imbalances that may affect patient engagement.

Similarly, synthesising assessment findings and making clinical judgements (C2.1.7) requires an appreciation of patient values, cultural identity, and capacity for self-care. Preventive and restorative plans may not always be practical or acceptable within a patient's circumstances.^20^ Cultural humility encourages shared decision-making, flexibility, and realistic goal setting, supporting care plans that are both clinically appropriate and achievable.

Understanding and managing the impact of medical and psychological conditions (C2.2.1) further reinforces the importance of cultural humility. Conditions such as anxiety, depression, substance use, neurodiversity, or chronic illness can significantly affect attendance, communication, and self-care behaviours. Cultural beliefs and stigma surrounding mental health or illness may shape how patients perceive diagnosis and treatment, influencing engagement and adherence. For example, recent research has found a dual frontier between mental and oral health: patients with anxiety and depression are less likely to seek or adhere to dental care despite dental health issues, such as periodontitis, worsening with anxiety and depression.^21^^,^^22^ Similarly, neurodiverse individuals may face additional challenges accessing and adhering to dental care due to lack of societal and clinical adaption to their needs.^23^ Trainees must learn to recognise these interactions and adapt their management accordingly, rather than imposing standardised approaches and attributing non-compliance to choice alone.

Community-based or outreach learning experiences are particularly effective in developing these competencies; examples are provided in Table 1.^24^^,^^25^^,^^26^^,^^27^ Exposure to diverse patient populations and healthcare settings enables students to appreciate how social and structural factors influence oral health behaviours, access to care, and treatment outcomes. Such experiences encourage reflection, challenge assumptions, and support the development of more responsive and patient-centred approaches to care. These experiences also support social accountability as they encourage students to want to work across all sectors of their community in their future dental careers.^28^

By embedding cultural humility into clinical and technical skills, dental education supports the development of practitioners who are not only technically competent, but also reflective, adaptable, and responsive to the needs of diverse populations.

### Domain: Interpersonal skills

#### *Sub-domain: Teamwork and wellbeing of others*

*I2.4: Explain the contribution that team members and effective team working makes to the delivery of safe and effective high-quality care, including the benefits of working in culturally diverse teams.*

Effective teamwork is central to safe and high-quality dental care, and culturally diverse teams offer important benefits for patient experience, problem-solving, and innovation. Embedding cultural humility within teamwork supports both learner wellbeing and the development of inclusive professional behaviours.

Understanding the contribution of team members and effective team working (I2.4) requires trainees to recognise the value of different perspectives, backgrounds, and lived experiences within the dental team. Culturally diverse teams can enhance communication with patients, improve cultural understanding, and challenge unconscious bias in clinical decision-making. However, these benefits are only realised when teams operate within a culture of respect, psychological safety, and mutual accountability.

Dental education programmes can support this by embedding structured opportunities for dialogue, reflection, and collaborative learning that promote cultural awareness, inclusive communication and respect for differences. Educational approaches that encourage students to explore cultural identity, recognise the strengths of diversity, and challenge exclusionary behaviours can strengthen teamwork and contribute to safer patient care; examples of such initiatives are outlined in Table 1.^29^^,^^30^

By learning to work effectively within culturally diverse teams, dental education can prepare trainees to contribute positively to different clinical environments, support colleagues' wellbeing, and deliver safer, more inclusive care to diverse patient populations.

### Domain: Professionalism

#### *Sub-domain: Ethics and integrity*

*P(B)3: Demonstrate cultural competence, accepting and respecting the diversity of patients and colleagues*

*P1.4: Explain cultural competence and its relevance in assessing the needs and planning care for patients from diverse backgrounds.*

Professionalism in dentistry requires a commitment to ethical practice, integrity, and respect for diversity. Cultural humility is a core component of ethical professionalism, enabling trainees to recognise and respect the limits of their own perspectives and respond appropriately to the needs of patients and colleagues from diverse backgrounds.

Demonstrating cultural competence (P(B)3) involves accepting and respecting diversity in all professional interactions. This includes acknowledging differences in culture, belief, identity, and lived experience without stereotyping or judgement. Cultural humility strengthens this competence by encouraging ongoing self-reflection, learning, and openness, rather than assuming static knowledge of ‘other' cultures.

Understanding cultural competence and its relevance to assessing needs and planning care (P1.4) is particularly important in training. Ethical patient care requires practitioners to consider how cultural identity, social context, and structural inequalities shape health needs and access to care. Without this awareness, well-intentioned care may inadvertently exclude or disadvantage certain groups.

Experiential, community-based and co-produced educational activities can support students in exploring culture, identity, marginalisation and healthcare access through the lens of lived experience. Such approaches encourage empathy, critical reflection, and a deeper appreciation of the ethical responsibilities associated with delivering equitable care; examples are summarised in Table 1.^31^^,^^32^^,^^33^

By embedding cultural humility within ethics and integrity education, dental training supports the development of reflective, respectful, and socially accountable professionals who are equipped to deliver equitable care in a diverse society.

These case studies, in Table 1, are examples of good practice that align with the GDC's *SPF* to create the future dental professionals. This is not an exhaustive list of methods to achieve these behaviours and learning outcomes. Whilst teaching our future dental professionals, it is also important that educators should reflect on their own cultural competence and be aware that none of us will ever be fully competent in culture owing to its ever-evolving nature. The authors note that whilst these case studies are examples of good practice in the current climate and context, this may change as opinions and ideas adapt in the future.

## A practical starting point for educators

For educators seeking to embed cultural humility within dental education, the breadth of potential approaches can appear overwhelming. However, meaningful change does not require whole curriculum redesign. Small, intentional changes can help create a more inclusive learning environment while supporting achievement of the GDC *SPF* outcomes. A practical starting-point checklist is provided in Table 2; this is intended as a starting point rather than a prescriptive framework. Institutions should adapt approaches according to local context, available resources, and the needs of their learners and communities.

**Table 2:** A practical starting-point checklist for educators

**1**	**Review existing curriculum content** Map current teaching activities against relevant SPF outcomes Identify where social determinants of health, cultural diversity, health inequalities, and protected characteristics are already discussed Consider opportunities to make cultural influences on oral health more explicit
**2**	**Develop foundational understanding** Ensure educators are familiar with key concepts including cultural humility, EEDI principles, unconscious bias, and the Equality Act 2010 Encourage staff reflection on their own assumptions, perspectives, and learning needs
**3**	**Incorporate lived experience** Include patient narratives, videos, case studies, or co-produced resources that represent diverse populations Where possible, involve patients and community members in curriculum development and delivery
**4**	**Embed reflection** Use reflective discussion, journalling, or facilitated debriefing following teaching sessions. Encourage students to consider how their own experiences and assumptions may influence patient care
**5**	**Create safe learning environments** Establish ground rules that promote respectful dialogue and constructive challenge Support students to explore uncertainty and ask questions without fear of judgement
**6**	**Expand learning beyond the clinical setting** Engage with community-based learning opportunities and outreach programmes Encourage students to explore how social and structural factors influence oral health and access to care
**7**	**Evaluate and continuously improve** Seek feedback from students, patients, and community partners Review initiatives regularly to ensure they remain relevant, inclusive, and responsive to changing populations and societal needs
EEDI, equality, equity, diversity and inclusion; SPF, *Safe practitioner framework*.	

The following resources may be helpful when developing cultural humility within healthcare education:

- GDC *SPF* Framework: https://www.gdc-uk.org/education-cpd/dental-education/quality-assurance/learning-outcomes-and-behaviours
- NHS England resources on Equality, Diversity and Inclusion https://www.leadershipacademy.nhs.uk/resources/inclusion-equality-and-diversity/
- Advance HE guidance on Equality, Diversity and Inclusion in Higher Education: https://www.advance-he.ac.uk/equality-diversity-and-inclusion
- Guidance relating to the Equality Act 2010: https://www.gov.uk/guidance/equality-act-2010-guidance
- Dental Schools Council resources relating to widening participation and inclusive education: https://www.dentalschoolscouncil.ac.uk/news/dental-schools-council-report-sets-out-roadmap-to-tackle-regional-inequalities-in-dental-education/
- Medical and Dental Schools Councils guidance on ‘Decolonising medical and dental curricula' which includes elements on cultural humility and inclusive approaches to education: https://www.medschools.ac.uk/latest/publications/decolonising-medical-and-dental-curricula-2/
- Local community and patient-partnership organisations that support co-production and lived-experience engagement.

Future work should focus on evaluating the impact of these educational approaches on student development, patient experience, and the reduction of oral health inequalities, while continuing to share examples of good practice across dental education programmes.

## Conclusion

Dental professional education has been updated with the GDC's *SPF* to include culture throughout key learning objectives. Through the provision of case studies, this article provides practice tools to support educators in embedding culture within their dental curriculum.
